# Common Colds

**Published:** 1862-07

**Authors:** J. Blackmer

**Affiliations:** Somerville


### Selections.

Common Colds.—*Messrs. Editors:*—Every one is practically familiar with common colds. The chilliness and shivering, the dullness and languor, the soreness of throat, pain in the head, stuffed nostril, and, still worse, the irritability of temper and the general discomfort, have not only been experienced by every one at some time, but it has been the misfortune of most people to pass through this unpleasant ordeal repeatedly and frequently.

It is certainly quite unnecessary for us to prepare an article upon the symptoms of a common cold, and although a consideration of the pathology and means of cure of this complaint might be interesting and profitable, I only propose at present to indicate what my own observation and experience appear to teach is a valuable and efficient preventive of this disorder. It is simply sulphuric ether. It should be taken by inhalation. A very little of the remedy will answer the entire purpose. The patient should not make an approach to etherization, but only apply the nose to a bottle containing the liquid and make a few inspirations. This must be done (in order to be completely effectual) when the first symptoms of the cold manifest themselves. The result is an almost immediate and complete removal of all the symptoms, and no unpleasant effect ensues. J. BLACKMER, M. D.

SOMERVILLE, June, 1862.

[*Boston Med. and Surg. Jour.*

